# Predicting Ki‐67 labeling index level in early‐stage lung adenocarcinomas manifesting as ground‐glass opacity nodules using intra‐nodular and peri‐nodular radiomic features

**DOI:** 10.1002/cam4.4719

**Published:** 2022-03-24

**Authors:** Minghui Zhu, Zhen Yang, Wei Zhao, Miaoyu Wang, Wenjia Shi, Zhenshun Cheng, Cheng Ye, Qiang Zhu, Lu Liu, Zhixin Liang, Liangan Chen

**Affiliations:** ^1^ Department of Respiratory Medicine Chinese People's Liberation Army General Hospital Beijing China; ^2^ Department of Pulmonary and Critical Care Medicine Zhongnan Hospital of Wuhan University Wuhan Hubei China; ^3^ Department of Immunology University of Pittsburgh School of Medicine Pittsburgh Pennsylvania USA; ^4^ Department of Nutrition, Chinese People's Liberation Army General Hospital Beijing China

**Keywords:** ground‐glass opacity nodule, Ki‐67, lung cancer, machine learning, radiomics

## Abstract

**Objectives:**

To explore the diagnostic value of radiomics in differentiating between lung adenocarcinomas appearing as ground‐glass opacity nodules (GGO) with high‐ and low Ki‐67 expression levels.

**Materials and Methods:**

From January 2018 to January 2021, patients with pulmonary GGO who received lung resection were evaluated for potential enrollment. The included GGOs were then randomly divided into a training cohort and a validation cohort with a ratio of 7:3. Logistic regression (LR), decision tree (DT), support vector machines (SVM), and adaboost (AB) were applied for radiomic model construction. Area under the curve (AUC) of the receiver operating characteristic (ROC) curve was used to evaluate the diagnostic efficacy of the established models.

**Results:**

Seven hundred and sixty‐nine patients with 769 GGOs were included in this study. Two hundred and forty‐five GGOs were confirmed to be of high Ki‐67 labeling index (LI). In the training cohort, gender, age, spiculation sign, pleural indentation sign, bubble sign, and maximum 2D diameter of the nodule were found to be significantly different between high‐ and low Ki‐67 LI groups (*p* < 0.05), and spiculation sign and maximum 2D diameter of the nodule were further confirmed to be risk factors for Ki‐67 LI. The radiomic model established using SVM exhibited an AUC of 0.731 in the validation cohort, which was higher than that of the clinical‐radiographic model (AUC = 0.675). Moreover, radiomic model combining both intra‐ and peri‐nodular features showed better diagnostic efficacy than using intra‐nodular features alone (AUC = 0.731 and 0.720, respectively).

**Conclusions:**

The established radiomic model exhibited good diagnostic efficacy in differentiating between lung adenocarcinoma GGOs with high and low Ki‐67 LI, which was higher than the clinical‐radiographic model. Peri‐nodular radiomic features showed added benefits to the radiomic model. As a novel noninvasive method, radiomics have the potential to be applied in the preliminary classification of Ki‐67 expression level in lung adenocarcinoma GGOs.

## Graphical abstract

By nodule segmentation, feature selection, modeling, and validation, a radiomic model was established to differentiate between lung adenocarcinomas appearing as ground‐glass opacity nodules with high‐ and low Ki‐67 expression levels, which exhibited good diagnostic efficacy.

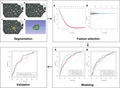

## 1 INTRODUCTION

Due to the changes of tobacco manufacturing and the growing attention to tobacco cessation worldwide, the incidence of squamous cell lung cancer has decreased, while adenocarcinoma has emerged to be the predominant pattern of lung cancer and this trend is still rising.^1^, ^2^ Unlike small cell lung cancer or squamous cell lung cancer, early‐stage lung adenocarcinomas often appear as ground‐glass opacity nodules (GGO) which are defined as hazy opacity that have a lower density than the surrounding soft tissue structures in pulmonary computerized tomography (CT) images.^3^, ^4^ The general prognosis of lung adenocarcinoma is poor, however, if diagnosed and evaluated clearly at the early stage, the patients could still have a favorable outcome. Hence, it is important to discover new approaches that could improve the diagnosis and prognostic evaluation of early‐stage lung adenocarcinoma.

Ki‐67 protein, encoded by the *MKI67* gene located on chromosome 10q25‐ter, was first identified as an antigen in Hodgkin lymphoma cell nuclei.^5^ Previous studies have found that Ki‐67 is associated with ribosomal RNA transcription.^6^ Given that Ki‐67 is expressed in G1, S, and G2 phases of the cell circle but is absent in the G0 phase quiescent cells, it is regarded as an ideal indicator for cell proliferation.^7^ Typically, the expression level of Ki‐67 protein can be represented by the Ki‐67 labeling index (LI), which refers to the percentage of Ki‐67‐positive nuclei under the microscope in immunohistochemical (IHC) tests. Researches have revealed that Ki‐67 were highly expressed in malignant tissue, which was of diagnostic value in differentiating malignant from benign lesions.^8^, ^9^ Moreover, Ki‐67 LI was also correlated with tumor differentiation, metastasis, clinical stage, and survival rate of patients in different types of malignancies including lung adenocarcinoma, making it a potential prognostic biomarker.^10^, ^11^, ^12^, ^13^ Therefore, monitoring Ki‐67 LI in tumor tissue is meaningful in the prognosis evaluation of patients with lung adenocarcinoma. However, at present the Ki‐67 protein level is mostly assessed by IHC methods, which is sometimes unavailable due to lack of tumor samples. Hence, a noninvasive evaluation method for Ki‐67 expression level is needed to illustrate the prognostic state of lung adenocarcinoma.

Radiomics refers to the high‐throughput extraction of large amounts of quantitative data from medical images.^14^ The extracted data could then be used for disease diagnosis, patient management, and prognostic evaluation. A standard radiomic workflow includes delineation of the region of interest (ROI), extraction and selection of radiomic features, and establishment of the radiomic model.^15^ Because of the high contrast resolution between the pulmonary nodules and lung parenchyma which makes nodules easily delineated from adjacent lung tissue, radiomics has been considered as a suitable tool in pulmonary nodule assessment.^16^ Radiomics has been used in the diagnosis, subtype differentiation, prediction of anticancer therapies and prognosis of lung adenocarcinomas,^17^, ^18^, ^19^, ^20^ however, it has been rarely used in the evaluation of Ki‐67 LI of lung adenocarcinoma. The only research we found using radiomic features to predict Ki‐67 LI was limited by its small sample size and single modeling method, which could influence the efficacy and reproducibility of the established model.^21^ Moreover, the potential of peri‐nodular radiomic features on Ki‐67 LI prediction has not been explored yet. Therefore, we carried out this retrospective study with large sample size, numerous radiomic features, and various modeling methods to investigate the potential of radiomics on Ki‐67 LI prediction.

## 2 MATERIALS AND METHODS

### 2.1 Study population

**FIGURE 1:**
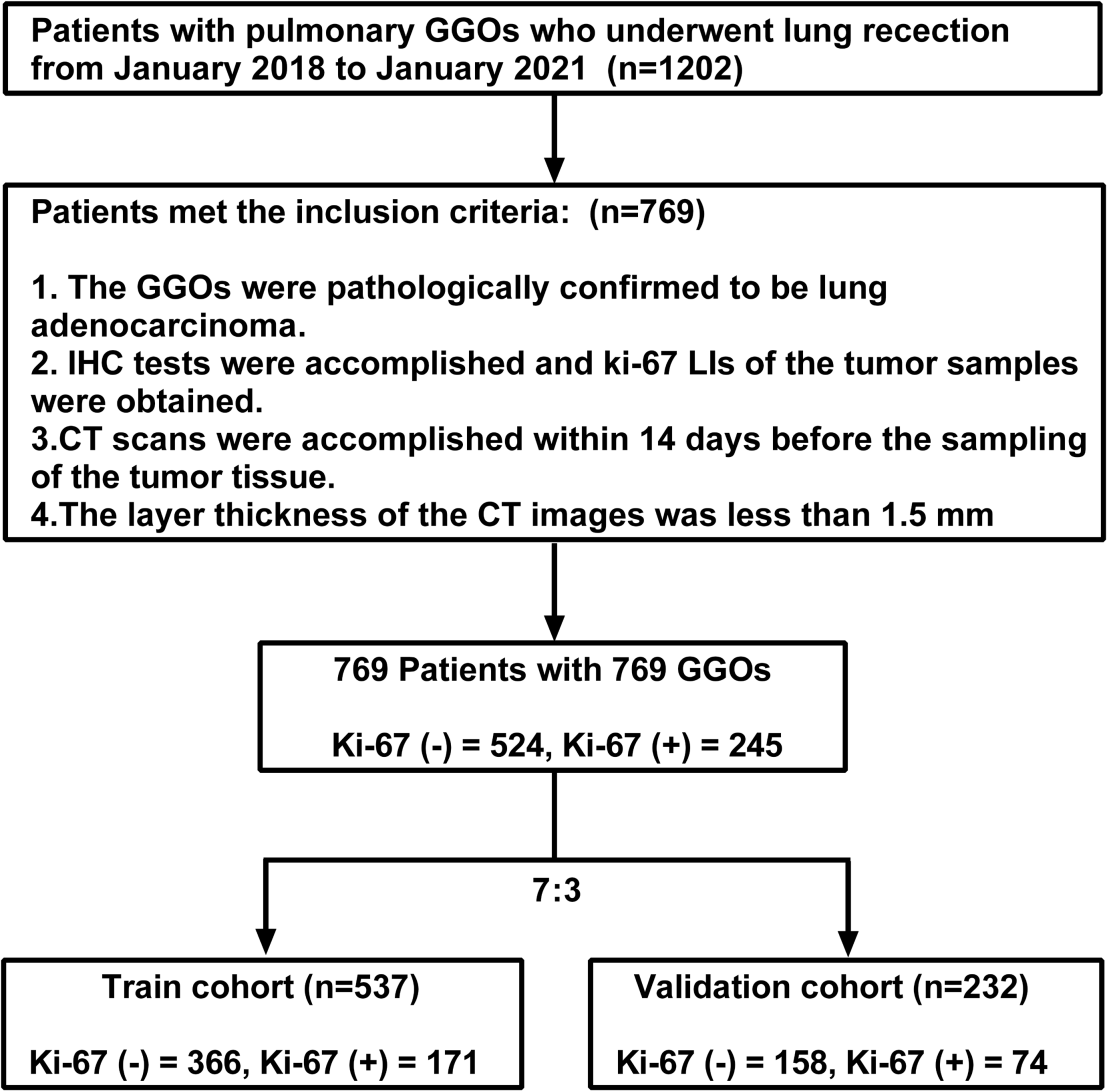
Flow chart of the study. Ki‐67 (−) refers to low Ki‐67 LI and Ki‐67 (+) refers to high Ki‐67 LI

Our institutional committee of ethics approved this retrospective study, and waived the requirement for informed consent of the patients. From January 2018 to January 2021, patients with pulmonary GGO who received segmentectomy, wedge resection, or lobectomy of lung in Chinese People's Liberation Army General Hospital were evaluated for enrollment. The inclusion criteria for this study were as followed: (1). The GGOs were pathologically confirmed to be lung adenocarcinoma; (2). IHC tests were accomplished and Ki‐67 LIs of the tumor samples were obtained; (3). CT scans were accomplished within 14 days before surgery; (4). The layer thickness of the CT images was less than 1.5 mm. Of note, for patients with multiple pulmonary GGOs, only those with confirmed pathological results were evaluated for possible enrollment. In total, 1202 patients were evaluated for enrollment and 769 patients with 769 GGOs were included in this study. The demographic and clinical characteristics of the patients were collected through our institutional medical record system. The GGOs were then randomly divided into a training cohort (*n* = 537) and a validation cohort (*n* = 232) with a ratio of 7:3. The workflow of this study is shown in Figure 1.

### 2.2 Data rebalance

Machine learning algorithms assume that the distribution of data in different groups are similar. If the data are imbalanced, machine learning algorithms tend to emphasize on the major class of the groups, which could lead to decrease of model performance. In this study, synthetic minority over‐sampling (SMOTE) method was used in the training cohort to rebalance the distribution of radiomic data and minimize the influence of class imbalance.

### 2.3 CT scanning parameters

All CT scanning were performed in one of the following scanners: Brilliance iCT (Phillips Medical Systems) or Somatom Definition (Siemens Medical Systems). The detailed CT scanning parameters are shown in Table 1.

**TABLE 1:** Parameters of the CT scanners

Parameters	Brilliance iCT	Somatom definition
Tube voltage (KV)	120	120
Tube current (mA)	110	110
Collimation	0.625 mm × 128	0.75 mm × 128
Pitch	1	1
Slice thickness (mm)	1	1.25
Slice interval (mm)	1	1.25
Rotation time (s)	0.5	0.5
FOV (mm)	364 × 364	364 × 364
Matrix size	512 × 512	512 × 512
Reconstruction kernel	iDose 3	B70f

### 2.4 Ki‐67 LI assessment procedure

Two experienced pathologists evaluated the Ki‐67 LI of the tumor samples of the patients. IHC test was carried out according to the manufacturer's instructions. In brief, formalin‐fixed tumor tissue samples were first embedded by paraffin and cut into 5 μm slices. Next, the slices were dried, dewaxed, rinsed in ethanol, and hydrated in H_2_0. Ki‐67 antibody was then used for IHC staining and cells with brown nuclei were considered positive. Finally, three areas with the most positive cells under high magnification microscope were chosen for Ki‐67 protein expression evaluation, and Ki‐67 LI was calculated by averaging the percentages of Ki‐67 positive cells in the chosen areas. According to previous researches^22^, ^23^ and the data distribution in this study, negative Ki‐67 level was considered as Ki‐67 LI < 10% and positive Ki‐67 level was considered as Ki‐67 LI ≥10%.

### 2.5 Evaluation of radiographic characteristics and building of the clinical‐radiographic model

Two physicians with 10 years and 5 years' experience in lung CT imaging, who were blinded to the pathological results reviewed the CT images and evaluated the radiographic characteristics of the GGOs. Group discussion would be held to reach a consensus if the two physicians had any disagreement. The collected radiographic characteristics are listed in Table 2.

**TABLE 2:** Clinical and radiographic features of the patients

Variables	Training cohort			Validation cohort		
	Ki‐67 (−) (*n* = 366)	Ki‐67 (+) (*n* = 171)	*p* value	Ki‐67 (−) (*n* = 158)	Ki‐67 (+) (*n* = 74)	*p* value
Gender						
Male	130 (35.5)	78 (45.6)	0.025	51 (32.3)	24 (32.4)	0.980
Female	236 (64.5)	93 (54.4)		107 (67.7)	50 (67.6)	
Age (years, average ± SD)	54.5 ± 9.1	56.6 ± 9.0	0.013	54.6 ± 10.0	55.7 ± 9.6	0.389
Having respiratory symptoms						
Yes	40 (10.9)	19 (11.1)	0.950	14 (8.9)	12 (16.2)	0.098
No	326 (89.1)	152 (88.9)		144 (91.1)	62 (83.8)	
BMI index	24.2 ± 2.9	24.4 ± 2.8	0.418	24.4 ± 3.2	23.9 ± 3.2	0.350
Smoking						
Yes	54 (14.8)	34 (19.9)	0.135	17 (10.8)	12 (16.2)	0.241
No	312 (85.2)	137 (80.1)		141 (89.2)	62 (83.8)	
Smoking index (pack‐year)	532.5 ± 377.0	659.3 ± 507.8	0.359	750.3 ± 623.5	614.6 ± 466.2	0.679
Former lung cancer history						
Yes	9 (2.5)	2 (1.2)	0.516	1 (0.6)	0 (0)	1.000
No	357 (97.5)	169 (98.8)		157 (99.4)	74 (100)	
Former malignancy history except lung cancer						
Yes	20 (5.5)	6 (3.5)	0.325	4 (2.5)	6 (8.1)	0.078
No	346 (94.5)	165 (96.5)		154 (97.5)	68 (91.9)	
Family history of lung cancer						
Yes	42 (11.5)	15 (8.8)	0.343	19 (12)	5 (6.8)	0.219
No	324 (88.5)	156 (91.2)		139 (88)	69 (93.2)	
Family history of malignancy except lung cancer						
Yes	65 (17.8)	20 (11.7)	0.073	20 (12.7)	13 (17.6)	0.318
No	301 (82.2)	151 (88.3)		138 (87.3)	61 (82.4)	
Abnormal tumor biomarker results						
Yes	59 (16.1)	24 (14)	0.533	19 (12)	12 (16.2)	0.382
No	307 (83.9)	147 (86)		139 (88)	62 (83.8)	
Multiple nodules						
Yes	177 (48.4)	82 (48)	0.930	75 (47.5)	38 (51.4)	0.581
No	189 (51.6)	89 (52)		83 (52.5)	36 (48.6)	
Nodule density						
Pure GGO	234 (63.9)	101 (59.1)	0.278	102 (64.6)	41 (55.4)	0.182
Mixed GGO	132 (36.1)	70 (40.9)		56 (35.4)	33 (44.6)	
Border						
Unclear	87 (23.8)	50 (29.2)	0.176	43 (27.2)	20 (27)	0.976
Clear	279 (76.2)	121 (70.8)		115 (72.8)	54 (73)	
Lobulation sign						
Yes	100 (27.3)	59 (34.5)	0.090	39 (24.7)	24 (32.4)	0.216
No	266 (72.7)	112 (65.5)		119 (75.3)	50 (67.6)	
Spiculation sign						
Yes	43 (11.7)	37 (21.6)	0.003	22 (13.9)	16 (21.6)	0.140
No	323 (88.3)	134 (78.4)		136 (86.1)	58 (78.4)	
Pleural indentation sign						
Yes	48 (13.1)	35 (20.5)	0.028	18 (11.4)	14 (18.9)	0.121
No	318 (86.9)	136 (79.5)		140 (88.6)	60 (81.1)	
Bubble sign						
Yes	56 (15.3)	40 (23.4)	0.023	23 (14.6)	12 (16.2)	0.742
No	310 (84.7)	131 (76.6)		135 (85.4)	62 (83.8)	
Vessel change						
Yes	83 (22.7)	45 (26.3)	0.357	25 (15.8)	16 (21.6)	0.280
No	283 (77.3)	126 (73.7)		133 (84.2)	58 (78.4)	
Maximum 2D diameter(mm, average ± SD)	12.6 ± 5.7	15.1 ± 6.0	<0.001	12.0 ± 5.2	15.4 ± 5.7	<0.001
Location						
Left upper lobe	86 (23.5)	45 (26.3)	0.497	44 (27.8)	23 (31.1)	0.726
Left lower lobe	53 (14.5)	28 (16.4)		28 (17.7)	10 (13.5)	
Right upper lobe	136 (37.2)	62 (36.3)		58 (36.7)	28 (37.8)	
Right middle lobe	17 (4.6)	11 (6.4)		6 (3.8)	5 (6.8)	
Right lower lobe	74 (20.2)	25 (14.6)		22 (13.9)	8 (10.8)	

*Note*: Ki‐67 (−) indicates low Ki‐67 LI and Ki‐67 (+) indicates high Ki‐67 LI.

Univariate analysis was performed to select the significant clinical and radiographic characteristics between Ki‐67 low‐ and high‐expression groups in the training cohort. Multivariate logistic regression was then performed to construct a clinical‐radiographic model. The area under the curve (AUC) of the receiver operating characteristic (ROC) curve was used to evaluate the performance of the established model.

### 2.6 Nodule segmentation, radiomic feature extraction, and intra/interobserver agreement evaluation

**FIGURE 2:**
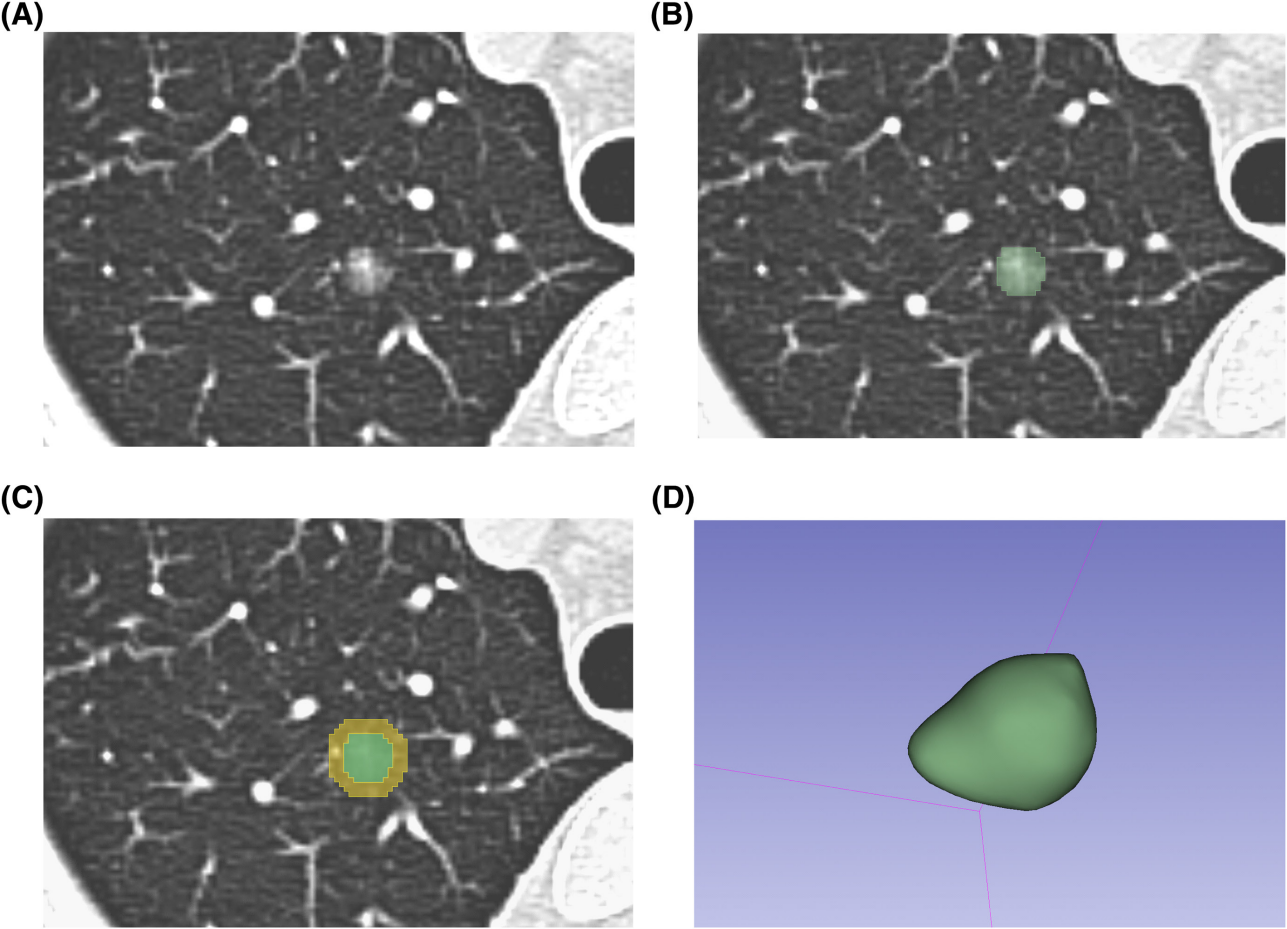
Segmentation process of the intra‐nodular and peri‐nodular regions in 3D slicer. (A) A ground‐glass opacity nodule (GGO) on the 1.5 mm CT slice. (B) Segmentation of the intra‐nodular region (marked green). (C) Peri‐nodular region was acquired by extending the intra‐nodular region by 5 mm from its boarder in 3D using function “Hollow” (marked yellow). (D) The 3D model of the delineated GGO

Intra/peri‐nodular segmentation was accomplished manually using 3D slicer software (version 4.10.2, https://www.slicer.org). Intra‐nodular region was obtained by delineating the outline of the GGOs. Peri‐nodular region was obtained by extending the intra‐nodular region by 5 mm from its boarder in three dimensions using function “Hollow” in 3D slicer (Figure 2).

The intra/peri‐nodular regions were then used for radiomic feature extraction via the “SlicerRadiomics” plug‐in in 3D slicer software (http://pyradiomics.readthedocs.io/). First, the images were resampled to 1 × 1 × 1 mm to minimize the influence of different CT reconstruction methods and body sizes of the patients. Then 1223 radiomic features including shape‐related, first order, texture, wavelet, and Laplacian of Gaussian (LoG) features were extracted from both intra‐nodular and peri‐nodular regions. Detailed list of the extracted radiomic features is shown in Appendix S1.

Intra/interobserver agreement was evaluated by calculating the intra/inter‐class correlation coefficient (ICC). Briefly, 60 randomly selected lung GGOs were delineated by two physicians (M.Z and Z.Y), respectively to assess the inter‐class correlation coefficient. One week later, the same 60 GGOs were then evaluated by M.Z again to calculate the intra‐class correlation coefficient. Both average ICC and ICC for each radiomic feature were calculated. Features with ICC <0.75 were removed due to lack of robustness.

### 2.7 Radiomic feature selection and building of the radiomic model

Least absolute shrinkage and selection operator (LASSO) method were used in this study for radiomic feature selection and dimensionality reduction to avoid potential over‐fitting of the established radiomic model. Tenfold cross‐validation method was used to find the optimal regularization parameter (*λ*) in which the LASSO model had minimum error.

Four machine learning methods, namely logistic regression (LR), decision tree (DT), support vector machines (SVM), and adaboost (AB), were used to construct four different models using the radiomic data in the training cohort, and the model with the best diagnostic performance in the validation cohort was chosen as the radiomic model. AUC was used to evaluate the diagnostic value of the established radiomic model.

### 2.8 Statistical analysis

R software (version 4.0.2, The Free Software Foundation) was used for statistical analysis and model construction in this study. Student's *t*‐test, Mann–Whitney *U* test or Pearson's *x* ^2^ test were applied for statistical significance evaluation according to the type and distribution of the data. Comparison between groups was considered statistically significant when *p* value was less than 0.05.

## 3 RESULTS

### 3.1 Clinical and radiographic characteristics of the patients

A total of 769 patients were included in this study, consisting of 486 females and 283 males. Among all the GGOs, 245 were confirmed to be of high Ki‐67 LI. In the high Ki‐67 LI group, the average age of the patients was 56.3 ± 9.2 years, which was significantly higher than that in the low Ki‐67 LI group (54.5 ± 9.4 years). The proportion of patients with respiratory symptoms and BMI indexes were alike in the high and low Ki‐67 LI groups. In the training cohort, gender, age, spiculation sign, pleural indentation sign, bubble sign, and maximum 2D diameter of the nodule were found to be significantly different between high and low Ki‐67 LI groups (*p* < 0.05). The detailed clinical and radiographic characteristics of the patients in the training cohort and validation cohort are presented in Table 2.

### 3.2 Building of the clinical‐radiographic model

In order not to exclude important factors, the clinical and radiographic factors with *p* < 0.1 between high and low Ki‐67 LI groups were included as candidates for subsequent modeling process. As shown in Table 2, the clinical and radiographic factors included were: gender, age, family history of malignancy except lung cancer, lobulation sign, spiculation sign, pleural indentation sign, bubble sign, and maximum 2D diameter of the nodule. Independent risk factors were further selected using forward stepwise multivariate logistic regression. As presented in Table 3, spiculation sign and maximum 2D diameter of the nodule were identified as independent risk factors and were incorporated into a clinical‐radiographic model using logistic regression. The AUCs of the clinical‐radiographic model in the training and validation cohort were 0.636 and 0.675, respectively (Figure 5).

**TABLE 3:** Multivariate logistic regression of the clinical‐radiographic features

Variables	*β*	OR (95% CI)	*p*
Intercept	−1.766	0.171	<0.001
Spiculation sign	0.563	1.757 (1.070–2.884)	0.026
Maximum 2D diameter	0.066	1.068 (1.035–1.103)	<0.001

### 3.3 Intra/interobserver agreement evaluation

The average ICC for intra and interobserver agreement evaluation were 0.90 and 0.89, respectively, which showed that the two researchers exhibited good consistency in nodule segmentation and feature extraction. In total, 442 features with ICC lower than 0.75 were removed, and the remaining 2004 features were used for further feature selection and model construction.

### 3.4 Feature selection, model performance, and comparison between clinical‐radiographic and radiomic models

**FIGURE 3:**
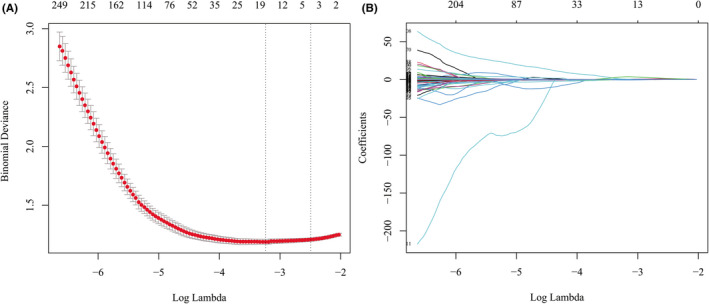
Feature selection using least absolute shrinkage and selection operator (LASSO). (A) When *λ* = 0.039, log *λ* = −3.244 (the first dotted vertical line), the model had minimum error, and 13 nonzero features were selected. (B) The coefficient profiles of the 2446 features

**FIGURE 4:**
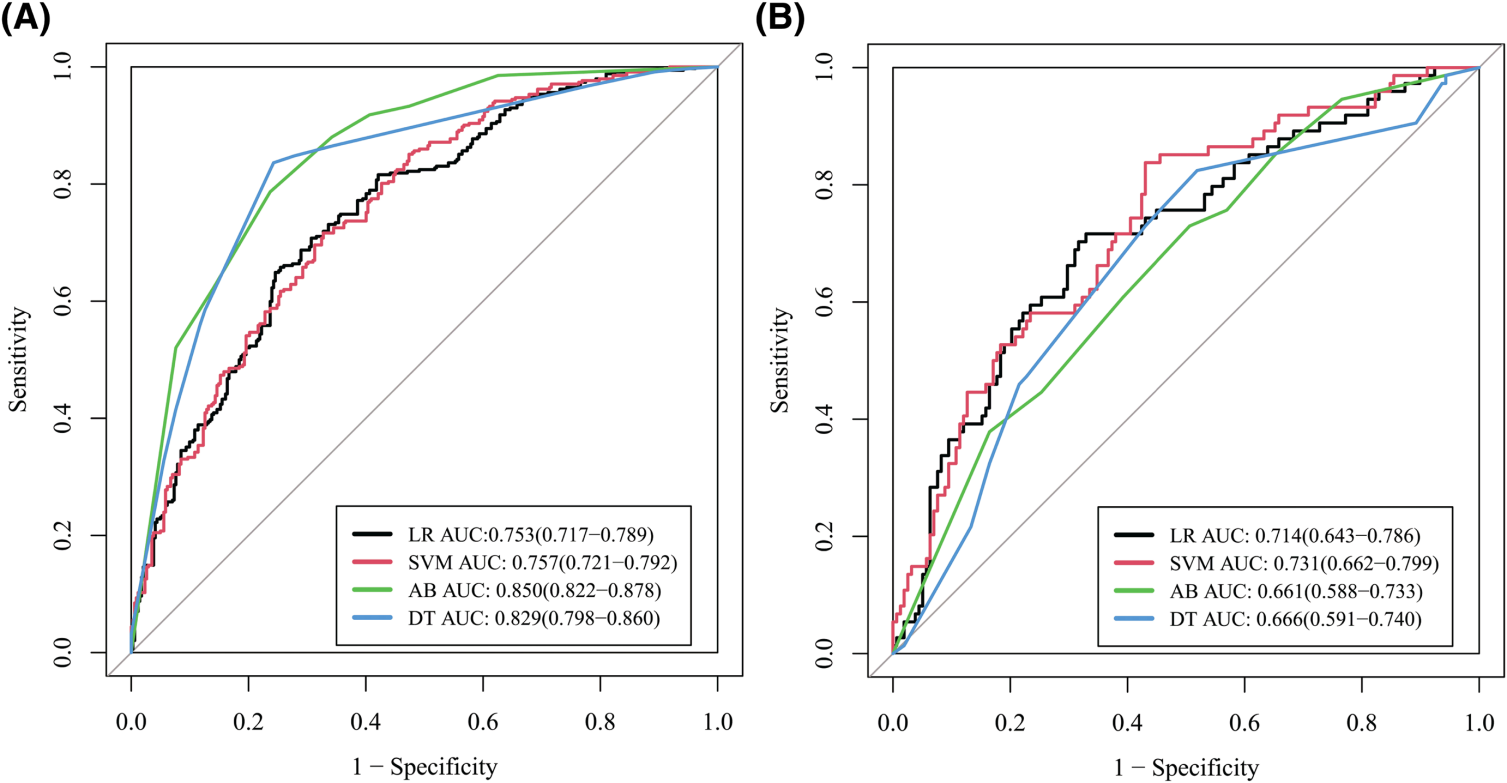
Receiver operating characteristic (ROC) curves of logistic regression (LR), support vector machine (SVM), decision tree (DT), and adaboost (AB) in the training (A) and validation (B) cohort. SVM exhibited the best performance (area under the curve (AUC) = 0.731) in the validation cohort. Data in the parentheses referred to the 95% confidence interval of AUC

**FIGURE 5:**
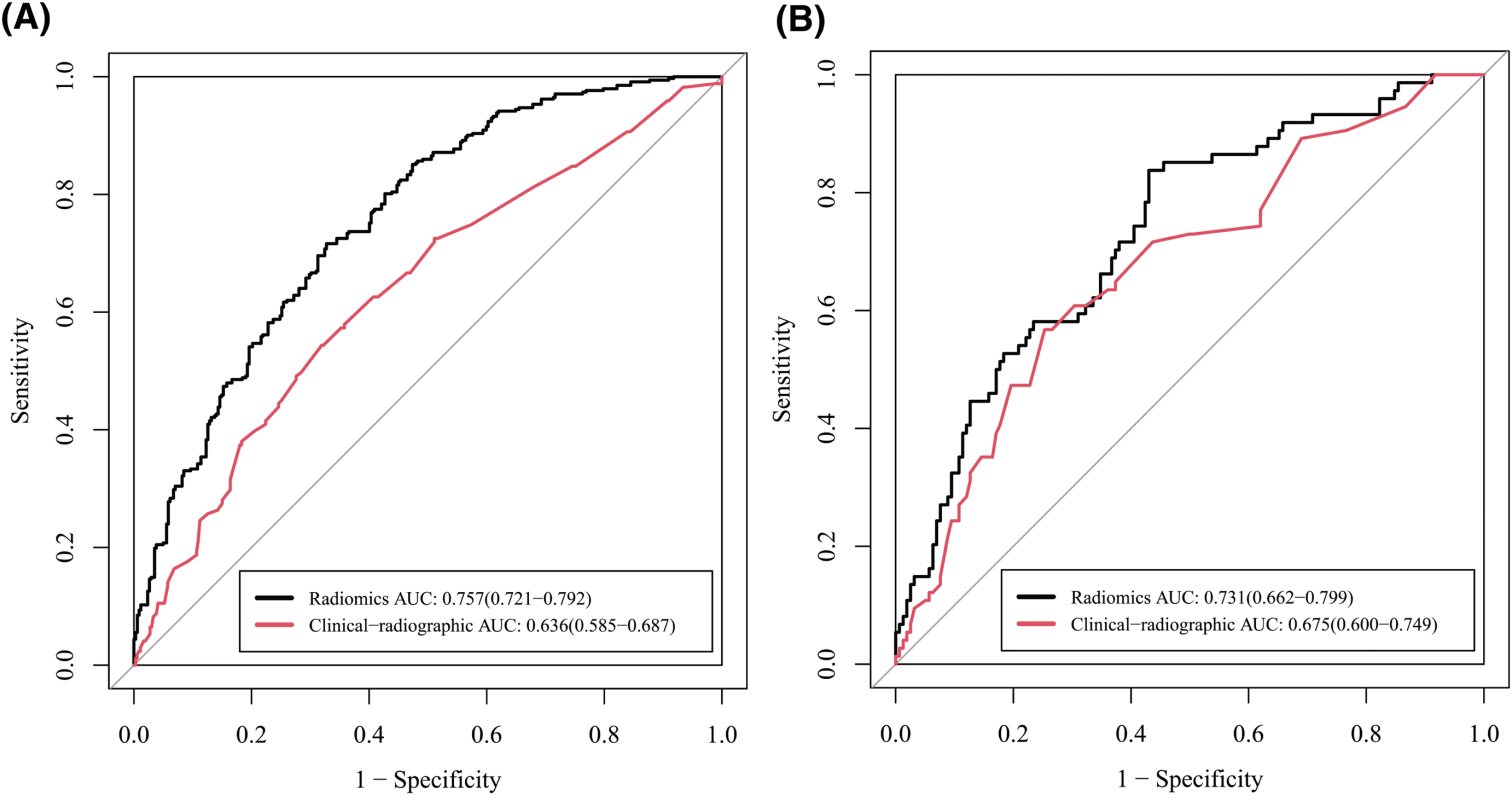
Receiver operating characteristic (ROC) curves of the clinical‐radiographic model and the radiomic model in the training (A) and validation (B) cohort. Data in the parentheses referred to the 95% confidence interval of area under the curve (AUC)

As shown in Figure 3, when *λ* = 0.039, log*λ* = −3.244, the LASSO model had the minimum error, and 13 radiomic features were selected. The selected radiomic features are listed in Appendix S1. LR, DT, SVM, and AB were used for model construction in both training and validation cohorts. SVM exhibited the best performance in the validation cohort among the four machine learning methods (Figure 4), therefore it was used to construct the radiomic model. The AUC of the radiomic model for identifying nodules with high Ki‐67 LI in the validation cohort was 0.731, which was significantly higher than that of the clinical‐radiographic model, indicating that the radiomic model had better diagnostic efficacy (Figure 5).

### 3.5 Diagnostic value of peri‐nodular features

**FIGURE 6:**
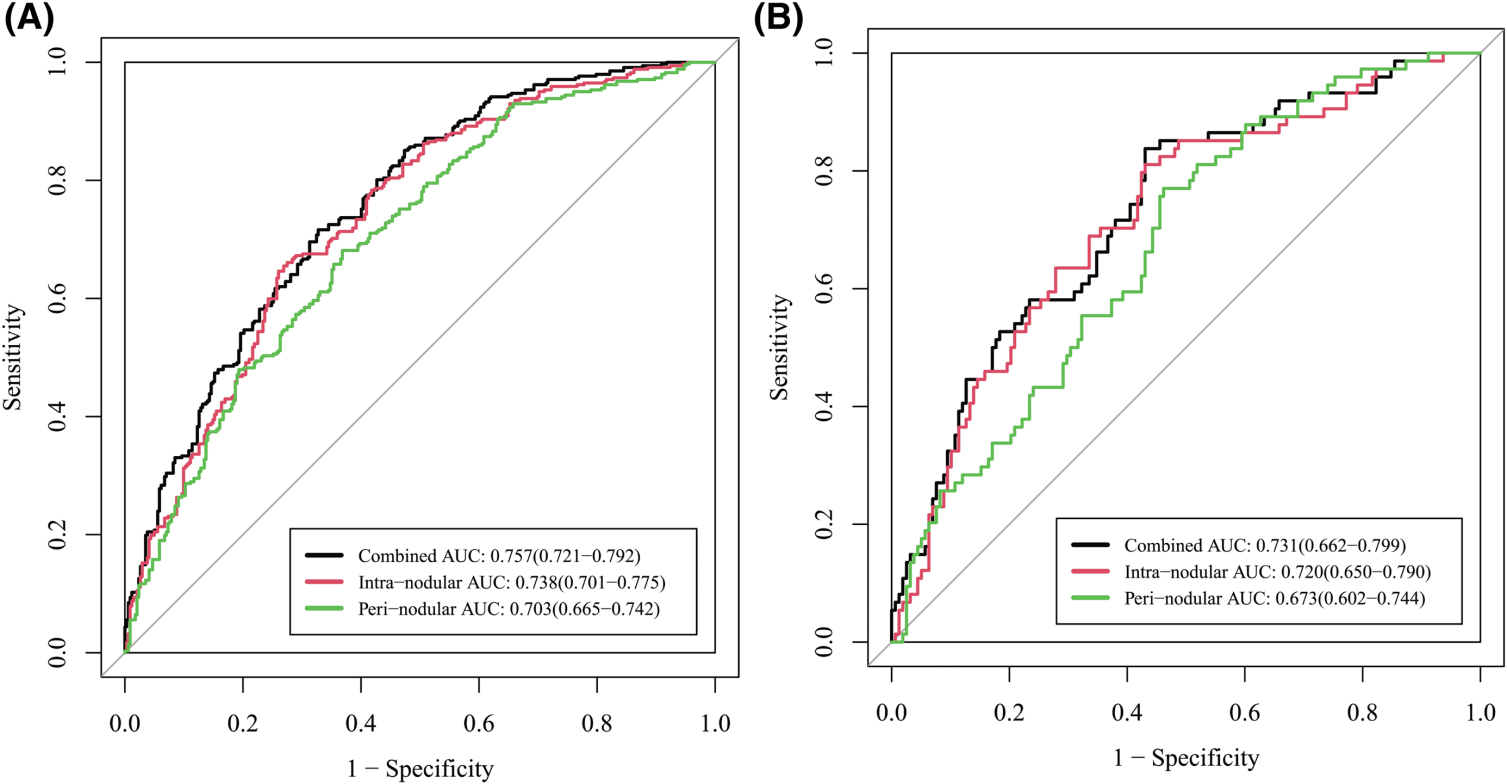
Receiver operating characteristic (ROC) curves of intra‐nodular, peri‐nodular, and combined radiomic models in the training (A) and validation (B) cohort. Data in the parentheses referred to the 95% confidence interval of area under the curve (AUC)

The diagnostic value of peri‐nodular radiomic features in differentiating between GGOs with high and low Ki‐67 LI was also tested in this study. As presented in Figure 6, the AUCs for intra‐nodular and peri‐nodular radiomic features in the validation cohort were 0.720 and 0.673, respectively. Combining intra‐ and peri‐nodular features yielded the best performance (AUC = 0.731), which showed that peri‐nodular radiomic features could help improve the diagnostic efficacy of radiomic model in differentiating between GGOs with high and low Ki‐67 LI.

## 4 DISCUSSION

The present study aimed to explore the diagnostic value of intra‐nodular and peri‐nodular radiomic features in differentiating between GGOs with high and low Ki‐67 LI. Using LASSO and four different machine learning methods (LR, DT, SVM, and AB), the constructed radiomic model outperformed the clinical‐radiographic model, indicating the potential use of radiomics in predicting Ki‐67 status in pulmonary GGOs.

Since the concept of “radiomics” was put forward in 2012, it has been used in various aspects of lung cancer such as the differentiation of benign and malignant nodules, and the evaluation of the invasiveness of pulmonary lesions.^24^, ^25^, ^26^ In recent years, radiomics has also been applied to predict the molecular patterns of lung cancer lesions such as driver gene mutations and PD‐L1 status, and yielded good diagnostic efficacy,^27^, ^28^, ^29^ indicating that differences at the molecular level could be possibly captured and described with radiomic features in radiographic images. However, radiomic studies concentrating on gene expression changes of lung cancer nodules were rarely seen, leaving room for further investigation. Ki‐67 is a commonly detected protein in routine IHC tests of samples acquired in lung resections. Highly expressed Ki‐67 often indicates rapid cell proliferation, which might lead to poor prognosis in cancer cells. Monitoring Ki‐67 level could be hard due to the inconvenience of frequent biopsy, hence a noninvasive radiomic method would be valuable. Therefore, in this study, we explored the value of radiomics in predicting Ki‐67 LI in lung adenocarcinoma GGOs.

Some clinical and radiographic characteristics were found to be related to the expression level of Ki‐67. Researchers found that Ki‐67 level correlated with tumor category and TNM stages.^13^ In a systematic review involving 108 studies and 14,732 lung cancer patients, higher Ki‐67 level was found to be related with age, male gender, smoking status, tumor size, and higher pathologic stages.^30^ In our study, we also found that age, male gender, and tumor diameter were related to Ki‐67 expression level, which was similar to former studies. Moreover, certain radiographic signs, namely spiculation sign, pleural indentation sign, and bubble sign, were also found to appear more frequently in GGOs with higher Ki‐67 levels. This could be partly explained by the fact that these radiographic signs always exist in CT images of lesions consisting of fast‐growing lung cancer cells, which often express high level of Ki‐67 protein. Further multivariate logistic regression revealed that spiculation sign and maximum 2D diameter of the GGO were risk factors for Ki‐67 level. However, the clinical‐radiographic model consisting of these two factors only exhibited weak diagnostic value in differentiating between Ki‐67‐high and low GGOs (AUC = 0.675 in validation cohort), indicating limited application in clinical practice.

We further tested the value of radiomics in predicting the Ki‐67 LI of lung adenocarcinoma appearing as GGOs. Four different machine learning methods were tested and SVM was found to have the best diagnostic efficacy. The AUC of the radiomic model reached 0.731 in the validation group, which was significantly higher than that of the clinical‐radiographic model. In another study, researchers found that three radiomic features (inverse variance, minor axis, and elongation) correlated with Ki‐67 level, and the AUC to identify Ki‐67 level for inverse variance was 0.770.^21^ However, the sample size of the research was small (*n* = 110), and there was no validation cohort to confirm the diagnostic efficacy of the established radiomic model, which might lead to an optimistic estimate. Moreover, we tested the diagnostic value of peri‐nodular radiomic features in predicting Ki‐67 level for the first time. As shown in Figure 6, it was noticeable that the combination of both intra‐ and peri‐nodular radiomic features outperformed using only intra‐nodular features. In clinical practice, peri‐nodular area was always ignored because the changes in this area were so tiny that they could hardly be captured by naked eyes. However, featured by high resolution, radiomics was able to identify tiny changes and hence could be used for data mining in peri‐nodular areas. Researches had discovered that peri‐nodular ring area might contain important information about micro vessels surrounding tumor area and was useful in predicting the invasiveness of lung adenocarcinoma lesions.^31^ In our study, we further confirmed that peri‐nodular radiomic features were also informative in predicting Ki‐67 level and should not be ignored in future studies.

Out of the 13 features incorporated in the radiomic model, nine of them were wavelet or LoG features. These features were acquired by applying filters to the original images so that some characteristics of the images such as edge area could be enhanced. Radiomic processing could work well on filtered images as well, however, one can hardly extract any information from a filtered image with naked eyes. This further explained why radiomic methods always outperformed routine image‐reading process. It is possible that future studies could further improve the diagnostic efficacy of radiomics with the discovery of more radiomic features and optimization of modeling processes.

This research has several limitations. First, in this single‐center retrospective study, a validation cohort had been set up to preliminarily verify the diagnostic efficacy of the established radiomic model. However, the conclusions of this study still need to be verified in other medical facilities and future prospective studies before clinical application. Second, patient follow‐up was not accomplished in this study so that prognostic analysis was unavailable. Therefore, multicenter prospective studies are still needed to settle the above issues.

In conclusion, the established radiomic model exhibited good diagnostic efficacy in differentiating between lung adenocarcinoma GGOs with high and low Ki‐67 LI, which was higher than the clinical‐radiographic model. Peri‐nodular radiomic features showed added benefits to the radiomic model. As a novel noninvasive method, radiomics has the potential to be applied in the preliminary classification of Ki‐67 expression level in lung adenocarcinoma GGOs.

## CONFLICT OF INTEREST

None declared.

## AUTHOR CONTRIBUTIONS

MZ and ZY collected, analyzed and interpreted the data, and wrote the manuscript. WZ, WS, and MW collected the data. ZC, CY, and QZ gave critical thoughts on the methods of the study and reviewed the manuscript. LL and ZL helped with the modeling process of the study. LC conceived the idea of the study, designed the workflow, and revised the manuscript.

## ETHICS STATEMENT

This retrospective study was approved by the ethics committee of First Medical Center of Chinese People's Liberation Army General Hospital, and the requirement for informed consent of the patients was waived.

## Supporting information


Appendix S1

## Data Availability

The datasets generated during the current study are not publicly available due to our institutional regulation, but are available from the corresponding author upon reasonable request.
