# Supplementary material for: Predicting Ki‐67 labeling index level in early‐stage lung adenocarcinomas manifesting as ground‐glass opacity nodules using intra‐nodular and peri‐nodular radiomic features
Source: Cancer Med. 2022 Mar 24;11(21):3982–92. doi: 10.1002/cam4.4719 (PMC9636499; doi:10.1002/cam4.4719)
Supplement: Supplementary file 1 — Appendix S1 [file CAM4-11-3982-s001.docx]

**Supplementary material**

**1 Supplementary methods**

**1.1 Radiomic features**

In this study, five groups of features were extracted: 1). Shape related features; 2). First order features; 3). Texture features; 4). Wavelet features; 5). Laplacian of Gaussian (LoG) features. A total of 2446 radiomic features were extracted for each GGO, including 1223 intra-nodular features and 1223 inter-nodular features.

**Group 1.** **Shape related features (n=14)**

Features in this group were used to describe the 2D and 3D shape and size of the delineated nodule. The included features were as followed: 1). Voxel Volume; 2). Maximum 3D Diameter; 3). Mesh Volume; 4). Major Axis Length; 5). Sphericity; 6). Least Axis Length; 7). Elongation; 8). Surface Volume Ratio; 9). Maximum 2D Diameter Slice; 10). Flatness; 11). Surface Area; 12). Minor Axis Length; 13). Maximum 2D Diameter Column; 14). Maximum 2D Diameter Row.

**Group 2.** **First order features (n=18)**

First order features describe the distribution of voxel intensities within the nodule region. Following features were included: 1). Interquartile Range; 2). Skewness; 3). Uniformity; 4). Median; 5). Energy; 6). Robust Mean Absolute Deviation; 7). Mean Absolute Deviation; 8). Total Energy; 9). Maximum; 10). Root Mean Squared; 11). 90 Percentile; 12). Minimum; 13). Entropy; 14). Range; 15). Variance; 16). 10 Percentile; 17). Kurtosis; 18). Mean.

**Group 3.** **Texture features (n=75)**

3.1 Gray Level Co-occurrence Matrix (GLCM) features (n=24)

GLCM describes the second-order joint probability function of an image region constrained by the mask, and provides information about the spatial orientation, distance and rangeability of voxel intensities. The GLCM features included in this study were: 1). Joint Average; 2). Sum Average; 3). Joint Entropy; 4). Cluster Shade; 5). Maximum Probability; 6). Inverse Difference Moment Normalized (Idmn); 7). Joint Energy; 8). Contrast; 9). Difference Entropy; 10). Inverse Variance; 11). Difference Variance; 12). Inverse Difference Normalized (Idn); 13). Inverse Difference Moment (Idm); 14). Correlation; 15). Autocorrelation; 16). Sum Entropy; 17). Maximal Correlation Coefficient (MCC); 18). Sum Squares; 19). Cluster Prominence; 20). Informational Measure of Correlation 2 (Imc2); 21). Informational Measure of Correlation 1 (Imc1); 22). Difference Average; 23). Inverse Difference; 24). Cluster Tendency.

3.2 Gray Level Run-Length Matrix (GLRLM) features (n=16)

GLRLM describes gray level runs of consecutive pixels that have the same gray level value. The extracted GLRLM features were: 1). Short Run Low Gray Level Emphasis; 2). Gray Level Variance; 3). Low Gray Level Run Emphasis; 4). Gray Level Non Uniformity Normalized; 5). Run Variance; 6). Gray Level Non Uniformity; 7). Long Run Emphasis; 8). Short Run High Gray Level Emphasis; 9). Run Length Non Uniformity; 10). Short Run Emphasis; 11). Long Run High Gray Level Emphasis; 12). Run Percentage; 13). Long Run Low Gray Level Emphasis; 14). Run Entropy; 15). High Gray Level Run Emphasis; 16). Run Length Non Uniformity Normalized.

3.3 Gray Level Size Zone Matrix (GLSZM) features (n=16)

GLSZM refers to the number of gray level zones in an image, and a gray level zone is defined as the number of connected voxels which have the same gray level value. The GLSZM features extracted were as followed: 1). Gray Level Variance; 2). Zone Variance; 3). Gray Level Non Uniformity Normalized; 4). Size Zone Non Uniformity Normalized; 5). Size Zone Non Uniformity; 6). Gray Level Non Uniformity; 7). Large Area Emphasis; 8). Small Area High Gray Level Emphasis; 9). Zone Percentage; 10). Large Area Low Gray Level Emphasis; 11). Large Area High Gray Level Emphasis; 12). High Gray Level Zone Emphasis; 13). Small Area Emphasis; 14). Low Gray Level Zone Emphasis; 15). Zone Entropy; 16). Small Area Low Gray Level Emphasis.

3.4 Gray Level Dependence Matrix (GLDM) features (n=14)

GLDM describes the number of connected voxels within a certain distance which are dependent on the center voxel. The included GLDM features were: 1). Gray Level Variance; 2). High Gray Level Emphasis; 3). Dependence Entropy; 4). Dependence Non Uniformity; 5). Gray Level Non Uniformity; 6). Small Dependence Emphasis; 7). Small Dependence High Gray Level Emphasis; 8). Dependence Non Uniformity Normalized; 9). Large Dependence Emphasis; 10). Large Dependence Low Gray Level Emphasis; 11). Dependence Variance; 12). Large Dependence High Gray Level Emphasis; 13). Small Dependence Low Gray Level Emphasis; 14). Low Gray Level Emphasis.

3.5 Neighborhood Gray Tone Difference Matrix (NGTDM) features (n=5)

NGTDM describes the difference between a gray value and the average gray value of its neighbors within a certain distance. 5 NGTDM features were collected in this study: 1). Coarseness; 2). Complexity; 3). Strength; 4). Contrast; 5). Busyness.

**Group 4. Wavelet features (n=744)**

Wavelet features were obtained by performing wavelet transforms to the original images. By applying filters (low-pass or high-pass) in three dimensions to the original image, 8 transformed images were acquired. If the original image was defined as X, H referred to a high-pass filter and L referred to a low-pass filter, then the 8 transformed images could be marked as: X_HHH_, X_HHL_, X_HLH_, X_HLL_, X_LHH_, X_LHL_, X_LLH_, X_LLL_. The first order features and texture features extracted from the 8 transformed images were collected as wavelet features.

**Group 5. Laplacian of Gaussian (LoG) features (n=372)**

LoG filter is a kind of edge enhancement filter which highlights the areas of gray level change. To obtain a LoG image, Gaussian kernel was first used for image smoothing, which was then convolved by the Laplacian kernel to enhance edges within the region. In this study, 4 different filter width (0.5mm, 1mm, 1.5mm, 2mm) was used to obtain 4 transformed LoG images. Then the first order features and texture features were extracted in each of the generated LoG images respectively.

**1.2 Radiomic features selected by LASSO**

In this study, LASSO was used to The radiomic features that were used to build the radiomic model. The radiomic features were listed as below:

**Table S1**. Radiomic features selected by LASSO.

| No. | Feature name |
| --- | --- |
| 1 | Inner_original_shape_Flatness |
| 2 | Inner_log-sigma-0-5-mm-3D_ngtdm_Strength |
| 3 | Inner_log-sigma-1-5-mm-3D_ngtdm_Strength |
| 4 | Inner_wavelet-HLH_glcm_Imc2 |
| 5 | Inner_wavelet-HHH_glcm_MCC |
| 6 | Inner_wavelet-HHL_gldm_DependenceEntropy |
| 7 | Inner_wavelet-LLL_firstorder_Median |
| 8 | Inner_original_gldm_DependenceEntropy |
| 9 | Outer_original_shape_Elongation |
| 10 | Outer_wavelet-LLH_ngtdm_Strength |
| 11 | Outer_wavelet-LLH_ngtdm_Busyness |
| 12 | Outer_wavelet-HLH_glcm_Imc2 |
| 13 | Outer_original_firstorder_RootMeanSquared |

The “inner/outer” refers to whether the feature is an intra-nodular or a peri-nodular feature.
